# Feasibility of face mask spirometry during decannulation in head and neck surgery: Prospective cohort study

**DOI:** 10.1111/coa.13938

**Published:** 2022-05-18

**Authors:** José Antonio Sánchez‐Guerrero, Maria Àngels Cebrià i Iranzo, Francisco José Ferrer‐Sargues, Sophie Périé

**Affiliations:** ^1^ Department of Otolaryngology Head Neck Surgery Faculty Medicine, Assistance Publique Hôpitaux Paris (APHP), Sorbonne University Paris France; ^2^ Department of Physiotherapy Universidad Cardenal Herrera‐CEU, CEU Universities Alfara del Patriarca Spain; ^3^ Department of Physiotherapy Universitat de València Valencia Spain; ^4^ Rehabilitation and Physical Medicine Service Hospital Universitari i Politècnic La Fe Valencia Spain; ^5^ Department of Otolaryngology Head and Neck Surgery Com Maillot ‐ Hartmann Clinic Neuilly Sur Seine France

**Keywords:** decannulation, face mask, head and neck cancer, spirometry, tracheostomy

## Abstract

**Objectives:**

To analyse the relationship between spirometric parameters measured with a face mask versus a mouthpiece, as well as the feasibility of face mask spirometric evaluation in a head and neck surgery (HNS) decannulation context. Furthermore, we examine peak inspiratory flow (PIF) cut‐off values before and after decannulation.

**Design:**

Prospective cohort study.

**Setting:**

Otolaryngology HNS Department of a university teaching hospital.

**Participants:**

Twenty‐four patients were selected. A maximal flow‐volume loop was conducted before (with mouthpiece) and after (with mouthpiece and face mask) decannulation.

**Main outcome measures:**

Recorded outcomes were forced vital capacity (FVC), forced expiratory volume in the first second, peak expiratory flow, PIF, forced expiratory flow at 50% of FVC and forced inspiratory flow at 50% of FVC. Spearman correlation coefficients between spirometric parameters measured with a face mask versus a mouthpiece were calculated. Wilcoxon test was used to check differences between mouthpiece and face mask values.

**Results:**

Correlation between mouthpiece and face mask spirometric values was moderate to high (*r* = 0.46–0.95). All parameters measured by spirometry were significantly lower with a face mask than those obtained with a mouthpiece (*p* < 0.05). Before decannulation, the lowest PIF value (tested with mouthpiece) that allowed successful decannulation was 1 L/s. After decannulation, the lowest PIF value tested with mouthpiece and face mask for successful completion of the decannulation process were 0.77 and 0.56 L/s, respectively.

**Conclusion:**

Face mask is a feasible option to perform a spirometry when face diseases hinder spirometric evaluation through a mouthpiece in an HNC surgery context.

**Key Points**

1. Face mask spirometry evaluates quantitatively the severity of upper airway obstruction in a routine clinical setting.
2. Face mask spirometry is an alternative strategy to evaluate patients with oral sphincter incompetence that prevents spirometric evaluation through a mouthpiece.
3. The PIF and PEF values obtained with a face mask were about 0.5 L/s lower than those obtained with a mouthpiece.
4. A PIF value of 1 L/s or higher, measured through a mouthpiece, was associated with a successful decannulation.
5. The airflow obstruction, caused by the cannula itself and revealed by spirometric evaluation, may have repercussions on the results of the capping trials.

## 1 OBJECTIVES

Temporary tracheostomy is commonly used after head and neck surgery in order to prevent severe upper airway obstruction (UAO) consequences, swallowing dysfunction or difficulties in managing secretions. However, tracheostomy remains a procedure associated with acute and late complications.^1^ In addition, tracheostomised patients need close monitoring and specialised care. Thus, as soon as physiological breathing is possible, decannulation is a desirable but challenging process.

Capping trial is the most used method prior to cannula removal. In this respect, published protocols recommend that decannulation is warranted after successful capping for 24–48 h.^2^

In recent years, a more functional approach to assess UAO with spirometry has been used at the bedside and in ambulatory patients before decannulation in head and neck cancer (HNC) patients after surgery.^3^, ^4^ Although some guidelines can be found in the literature,^3^, ^4^ spirometry cut‐off values must be optimised to decrease decannulation failure rates.

On the other hand, there are certain contexts such as limited mouth opening or neurological oral sphincter incompetence that prevent spirometric evaluation through a mouthpiece. Limited mouth opening after surgery can be the result of local resection, oedema, pain, temporo‐mandibular disorders or radiotherapy sequelae. Furthermore, neurological oral sphincter incompetence may be the consequence of transient or permanent damage of perioral nerves during surgery. Accordingly, face mask (FM) spirometry may be a good strategy to evaluate patients with these orofacial disorders. In this regard, the updated ATS/ERS spirometry guideline considers the use of FM in patients unable to use a mouthpiece.^5^

Until now, studies related to FM use focused their attention mainly on peak cough flow (in order to establish cough efficacy), forced vital capacity (FVC) and forced expiratory volume in 1 s (FEV_1_).^6^, ^7^, ^8^, ^9^ Peak inspiratory flow rate (PIF) has not been addressed. However, this parameter is currently used to evaluate upper airway obstruction and/or basis to determine the time of decannulation of patients after HNC surgery.^3^, ^4^, ^10^, ^11^, ^12^, ^13^

The aim of this study is to analyse the relationship between FM versus a mouthpiece spirometric values, as well as the feasibility of FM spirometric evaluation. Furthermore, we examine PIF cut‐off values before and after decannulation with mouthpiece and FM in an HNC surgery context.

## 2 DESIGN

A prospective cohort study was designed and conducted.

## 3 SETTINGS

The study was performed between November 2017 and January 2019 in the Otolaryngology Head and Neck Surgery Department of a university teaching hospital. The study was approved by the Commission d'Evaluation et de Recherche Observationnelle en OtoRhinoLaryngologie (CEROL) (Ethics Committee of the Society of Otolaryngology, France). In accordance with French legislation (Public Health Code amended by law no. 2004‐806, 9 August 2004 and the Huriet‐Sérusclat Law 88–1138, 20 December 1988), a patient information letter was issued. Data were strictly anonymous.

## 4 PARTICIPANTS

A total of 25 consecutive patients were eligible. Inclusion criteria were tracheostomised adults following elective surgery for HNC. Some of these patients stayed in the intensive care unit for a few days in order to monitor the free flap performed during surgery before discharge to HNC Surgery unit. Patients who failed to achieve at least three valid measurements before and after decannulation with mouthpiece and FM were excluded.

## 5 MAIN OUTCOME MEASURES

A maximal expiratory flow‐volume loop (FVL), which includes forced inspiratory manoeuvre, was conducted before and after decannulation according to the American Thoracic Society/European Respiratory Society (ATS/ERS) guidelines. Recorded outcomes were: FVC, FEV_1_, peak expiratory flow (PEF), PIF, forced expiratory flow at 50% of FVC (FEF 50%) and forced inspiratory flow at 50% of FVC (FIF 50%).

Measurements were performed with a portable spirometer (EasyOne 2001 diagnostic, ndd Medical Technologies). Before decannulation, 24 spirometric tests were performed with a mouthpiece (Spirette model 2050, ndd Medical Technologies) and a nose clip. After decannulation, measurements were performed with the mouthpiece (with a nose clip) and with a FM (Intersurgical Limited) connected to a sectioned mouthpiece (48 tests in all). A minimum of three FVLs were performed in each case before and after decannulation. For statistical analysis, FVLs with the best summative PEF‐PIF were selected.^4^

### 5.1 Decannulation and spirometric test protocols

Patients were tracheostomised with a non‐fenestrated cuffed tube (Shiley; Covidien plc). The cannula was usually changed the day after surgery for a fenestrated cuff‐less tracheostomy tube of the same diameter. Decannulation was commonly scheduled for the 3rd–6th day after surgery. The decision to decannulate was made by the physician following physical examination since the 3rd day after surgery, provided the PIF values were at least 1 L/s with the cannula still inserted. Decannulation was postponed in the presence of a medical complication (heart or respiratory failure, pneumonia or neurological complication) or early locoregional complications (haematoma or infections). In some cases, when decannulation could not be performed within the expected timeframes or when a maladjusted tracheostomy cannula was suspected, verification by fiberoscopy and/or spirometric assessment without cannula were also performed. Decannulation failure was defined as the need to recannulate the patient within 48 h of decannulation, whereas decannulation success was defined as the ability to continue breath without a tracheostomy with no significant dyspnoea.

Before decannulation, the FVC manoeuvre was obtained through the patient's mouth with the cannula (including the inner cannula) still inserted (and by obstructing the fenestrated cuff‐less tracheostomy tube) with a mouthpiece and a nasal clip. After decannulation, the FVC manoeuvre was obtained through a mouthpiece and FM (in both cases by obstructing the tracheostomy orifice). For FM spirometric tests, the patients were instructed to put the FM on their face and to perform the spirometric test to avoid air leaks. Decannulation and spirometry procedures were closely monitored by the same physical therapist, for each patient and with different time measurements. Moreover, to avoid the effects of fatigue or learning effects, after decannulation, the FM and mouthpiece spirometric tests were conducted in a random order.

### 5.2 Study size

The study population was a consecutive series of 24 patients who met the inclusion criteria and signed written informed consent. The study size had not been planned ahead. In any case, the recruitment process was declared completed prior to statistical analysis.

### 5.3 Statistical analysis

Data were analysed using XLSTAT (Version 2021.1) and BiostaTGV software program, provided by P. Louis (Epidemiologie‐Sante Publique, UMR S 1136). Continuous and categorical variables were expressed as means with standard deviations or percentages when appropriate for sample characteristics.

The planned statistical model could not be applied to the spirometric data because the Shapiro–Wilk test on the normality of some variables suggested that the statistical assumptions for applying Student's *t* test were not fulfilled. Thus, a non‐parametric test (Wilcoxon test) was adopted for use. Spirometric values and day of decannulation are presented descriptively, with the median selected as the most appropriate statistic given the skewness of the data.

Spearman's correlation coefficients were calculated in order to analyse the relationship between spirometric parameters measured with an FM versus a mouthpiece, and spirometric parameters measured with a mouthpiece before and after decannulation.

For the statistical analyses, a significance level was defined at *p* < 0.05.

## 6 RESULTS

### 6.1 Sample characteristics

**FIGURE 1:**
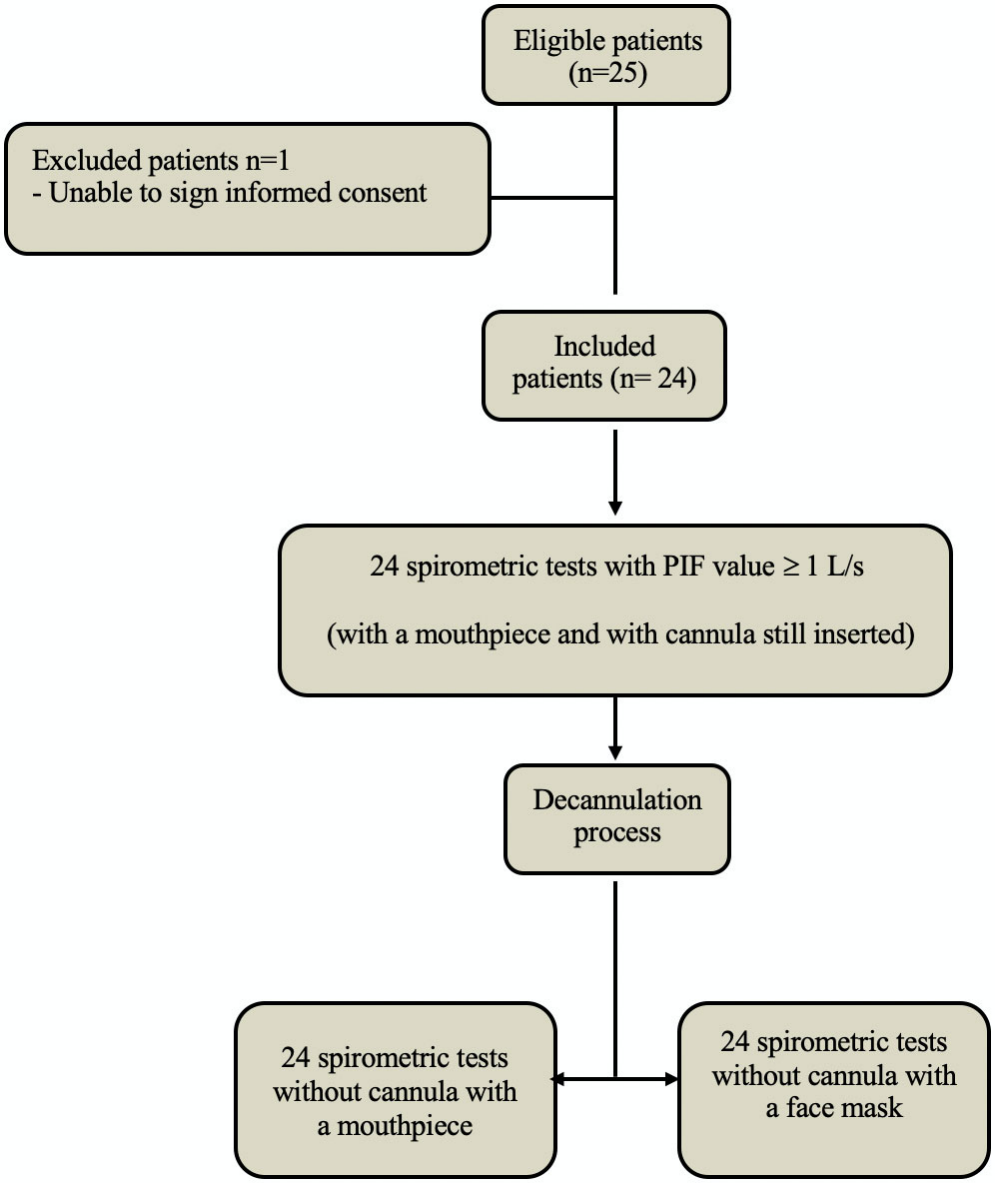
Flow diagram

Twenty‐five tracheostomised patients after HNC surgery were eligible during the study period (Figure 1). One patient was unable to give informed consent, consequently, 24 patients were finally analysed.

The age range for all the participants was between 39 and 79 years, and 75% were men (Table 1). The most frequent pathology was head and neck squamous cell carcinoma. Tracheostomy was performed during surgery for curative oncological surgery in 23 cases (with cervical lymph node dissection in 22) and for surgical drainage of cervical fasciitis in one case.

**TABLE 1:** Baseline characteristics of the patients with head and neck cancer (*n* = 24)

Anthropometrics	
Age (years)	62 ± 8.0
Sex (men/women)	18 (75%)/6 (25%)
Weight (kg)	70 ± 15.3
Height (cm)	169 ± 8.7
BMI (kg/m^2^)	24 ± 5
Smoking habit (yes, current or former)	21 (87%)
Type of tumour	
Head neck squamous cell carcinoma	22 (91%)
Ameloblastoma	1 (4%)
Not identified	1 (4%)
Surgery type	
Partial (pharyngo)laryngectomy	7 (29%)
Pelvi‐glossectomy with or without mandibulectomy	7 (29%)
Oropharyngectomy with or without mandibulectomy	8 (33%)
Rhinopharyngectomy	1 (4%)
Surgical drainage of cervical fasciitis	1 (4%)
Cervical neck dissection	
None	2 (8%)
Unilateral	16 (66%)
Bilateral	6 (25%)
Shiley cannula size (Number 8/Number 6)	18 (75%)/6 (25%)

*Note*: Values are expressed as mean ± 1 SD or number (percentage).

Abbreviation: BMI, body mass index.

The median time to decannulation was 7 days (3–67). Our decannulation failure rate was 0%.

### 6.2 Relationship between spirometric parameters measured with a face mask versus a mouthpiece after decannulation

Correlation between mouthpiece and FM spirometric values was moderate to high (*r* = 0.46–0.95) (Table 2). All parameters measured by spirometry were significantly lower with FM than with a mouthpiece (*p* < 0.05). Median PEF and PIF values with a mouthpiece were 4.06 and 2.06 L/s, respectively. Median PEF and PIF values with an FM were 3.56 and 1.42 L/s, respectively.

**TABLE 2:** Spirometric parameter values after decannulation obtained with a mouthpiece and a face mask (*n* = 24)

Variables	Mouthpiece	Face mask	*p* ^a^	*p* ^b^	*r*
FVC (L)	2.44 (0.89–4.18)	2.29 (0.68–4.06)	0.007	<0.001	0.951
FVC (% predicted)	71 (17–97)	52 (26–90)	<0.001	<0.001	0.918
FEV_1_ (L)	1.84 (0.67–3.46)	1.56 (0.59–3.94)	<0.001	< 0.001	0.923
FEV_1_(% predicted)	62 (41–94)	38 (16–79)	<0.001	<0.001	0.824
FEV_1_/FVC (%)	0.8 (0.5–1.0)	0.7 (0.5–0.9)	‐	‐	‐
PEF (L/s)	4.06 (2.17–7.77)	3.56 (1.13–6.05)	<0.001	0.001	0.769
PEF (% predicted)	54 (31–87)	Reference values not available			
FEF 50% (L/s)	2.09 (0.74–4.64)	1.47 (0.62–4.48)	0.022	0.004	0.569
FEF 50% (% predicted)	53 (21–102)	Reference values not available			
PIF (L/s)	2.06 (0.77–7.75)	1.42 (0.56–4.07)	<0.001	0.001	0.626
FIF 50% (L/s)	1.78 (0.58–7.60)	1.23 (0.35–3.96)	<0.002	0.013	0.502

*Note*: Values are expressed as median (minimum, maximum).

Abbreviations: FEF 50%, forced expiratory flow at 50% of FVC; FEV_1_, forced expiratory volume in the first second; FIF 50%, forced inspiratory flow at 50% of FVC; FVC, forced vital capacity; PEF, peak expiratory flow; PIF, peak inspiratory flow; *r*, Spearman correlation coefficients.

^a^ Wilcoxon signed‐rank test *p* values.

^b^ Spearman correlation *p* values.

### 6.3 Relationship between spirometric parameters before and after decannulation obtained with a mouthpiece

Correlation between PIF, FIF 50%, FEV_1_ and FVC values before and after decannulation was moderate to high (*r =* 0.575–0.81). Nevertheless, the correlation between FVC/FEV_1_, PEF and FEF 50% values before and after decannulation could not be demonstrated (*p* > 0.05). All parameters measured were significantly higher after decannulation (*p* < 0.05), except FEV_1_/FVC ratio and FIF 50% (Table 3).

**TABLE 3:** Spirometric parameter values before and after decannulation obtained with a mouthpiece (*n* = 24)

Variables	Before	After	*p* ^a^	*p* ^b^	*r*
FVC (L)	2.04 (0.55–4.14)	2.44 (0.89–4.18)	0.004	<0.001	0.810
FVC (% predicted)	49 (19–93)	71 (17–97)	0.001	<0.001	0.650
FEV_1_ (L)	1.38 (0.54–3.29)	1.84 (0.67–3.46)	<0.001	<0.001	0.792
FEV_1_ (% predicted)	46 (23–94)	62 (41–94)	<0.001	0.001	0.628
FEV_1_/FVC (%)	0.78 (0.44–0.99)	0.78 (0.52–1.0)	‐	‐	‐
PEF (L/s)	2.37 (1.21–6.67)	4.05 (2.17–7.77)	<0.001	0.111	0.334
PEF (% predicted)	34 (15–78)	54 (31–87)	<0.001	0.429	0.168
FEF 50% (L/s)	1.46 (0.01–4.75)	2.09 (0.74–4.64)	0.007	0.155	0.299
FEF 50% (% predicted)	36 (0–104)	53 (21–102)	0.007	0.197	0.272
PIF (L/s)	1.57 (1.01–5.36)	2.06 (0.77–7.75)	0.034	0.004	0.575
FIF 50% (L/s)	1.34 (0.29–4.84)	1.78 (0.58–7.60)	0.095	<0.001	0.646

*Note*: Values are expressed as median (minimum, maximum).

Abbreviations: FEF 50%, forced expiratory flow at 50% of FVC; FEV_1_, forced expiratory volume in the first second; FIF 50%, forced inspiratory flow at 50% of FVC; FVC, forced vital capacity; PEF, peak expiratory flow; PIF, peak inspiratory flow; *r*, Spearman correlation coefficients.

^a^ Wilcoxon signed‐rank test *p* values.

^b^ Spearman correlation *p* values.

### 6.4 Peak inspiratory flow cut‐off value with cannula for determining decannulation (measured through a mouthpiece)

**FIGURE 2:**
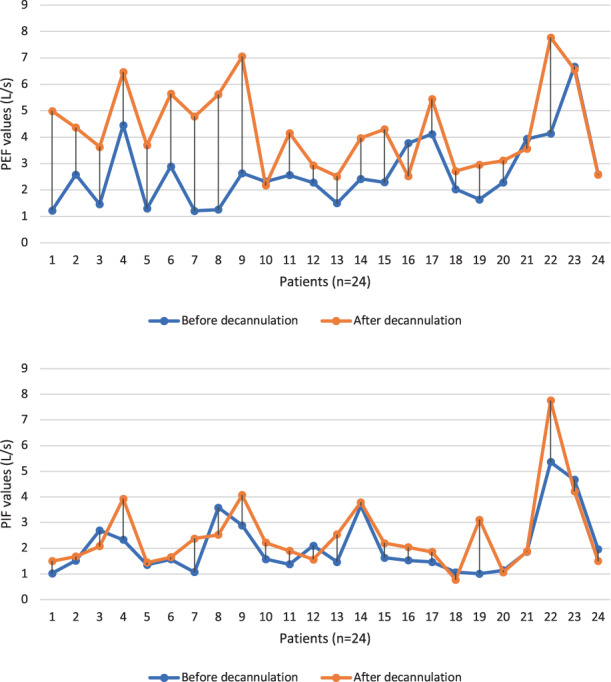
Peak expiratory flow (PEF, L/s) and peak inspiratory flow (PIF, L/s) values before and after decannulation using a mouthpiece (*n* = 24)

Median PIF value measured through a mouthpiece before decannulation was 1.57 L/s. The lowest PIF value before decannulation was 1.01 L/s. For all other parameters, see Table 2. Figure 2 shows the behaviour of PEF and PIF values before and after decannulation.

### 6.5 PIF cut‐off value without cannula for determining decannulation (measured through a mouthpiece and a face mask)

**FIGURE 3:**
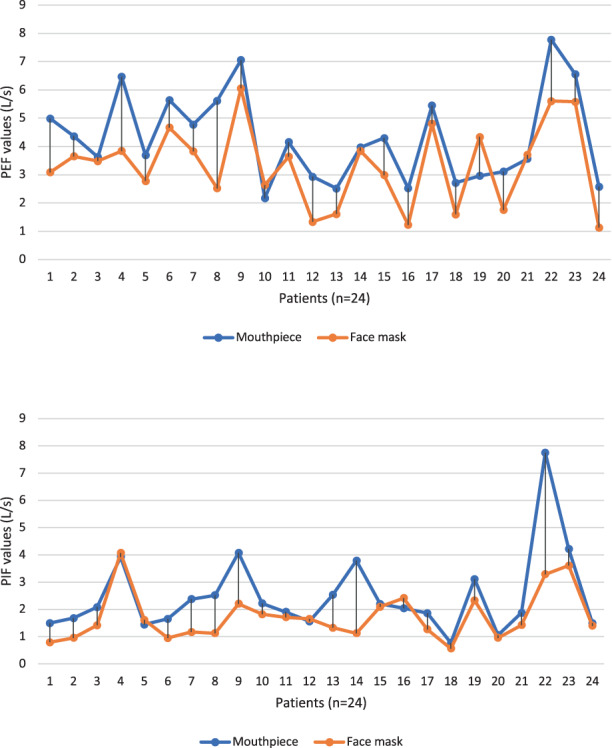
Peak expiratory flow (PEF, L/s) and peak inspiratory flow (PIF, L/s) values after decannulation with a mouthpiece and a face mask

Median PIF value measured through a mouthpiece after decannulation was 2.06 L/s. The lowest PIF value after decannulation was 0.77 L/s. Median PIF value measured through FM after decannulation was 1.42 L/s and the lowest PIF value that allowed successful decannulation was 0.56 L/s. For all other parameters through both interfaces, see Table 3. Figure 3 show the behaviour of PEF and PIF values with both interfaces after decannulation.

## 7 DISCUSSION

This study analysed the use of an FM for the evaluation of the upper airways in the decannulation process of patients in the context of HNC surgery. FM spirometric evaluation was feasible: all patients were easily evaluated with this interface and we cannot report any adverse event or difficulty worth highlighting. In accordance with previous studies, our results show that all spirometric values obtained with a mouthpiece were significantly higher than with an FM (*p* < 0.05).^7^ We can corroborate that the PIF and PEF values also follow this trend (Figure 3). Furthermore, a strong correlation has been shown between the PIF and PEF values measured with the two interfaces (*r* = 0.626 and *r* = 0.769, respectively). Our results reveal an average difference of 0.64 and 0.49 L/s between the PIF and PEF values obtained with an FM versus a mouthpiece. With regard to the values collected with the FM immediately after decannulation, minimum PIF and PEF values of 0.5 and 1.1 L/s allowed us to successfully decannulate all of our patients.

On the subject of PIF threshold for decannulation through a mouthpiece, in this sample, all patients could be decannulated with a PIF value of 1 L/s measured before decannulation. Current clinical guidelines assume a decannulation failure rate of 5%, similar to what we reported in a previous work using a PIF cut‐off value of 0.75 L/s (7%).^14^

Therefore, it seems that a threshold close to 1 L/s in HNC surgery context, as also proposed by Matyja^15^ may allow lower decannulation failure rates in accordance with the clinical guidelines.

It must be kept in mind that this recommended cut‐off value is given by tests with the cannula still inserted. Higher PIF and PEF values are expected after decannulation and have to be taken into account. In fact, when the obstruction of the airways is evaluated by spirometry with the cannula still inserted, the obstruction caused by the cannula itself is irremediably added.^4^ Moreover, the airway obstruction caused by the cannula has less impact on inspiratory parameters (a difference of around 0.5 L/s between PIF values before and after decannulation) than on expiratory ones (around 1.5 L/s between PEF values before and after decannulation), in line with our previous works (Figure 2).^4^

This airflow obstruction caused by the cannula itself, revealed by spirometric evaluation, may have repercussions on the results of the capping trials. To date, few researchers have addressed the issue.^16^, ^17^

### 7.1 Limitations and strengths

Although in this sample all patients could be decannulated at the first attempt, the reduced size of our study population prevents us from drawing final conclusions about the decannulation cut‐off.

A debatable aspect of our protocol is the absence of an FM test before decannulation. We consider that performing a spirometric evaluation with both interfaces before and after decannulation could overload patients.

Despite the small sample of our study, we believe it is representative of HNC patients in terms of age, gender, smoking habits and type of tumour.^18^

## 8 CONCLUSION

In HNC patients with limited mouth opening or oral neurological sphincter incompetence after surgery, using an FM to perform a spirometry is both feasible and reliable. A PIF value of 1 L/s or higher, measured through a mouthpiece, was associated with a successful decannulation.

## AUTHOR CONTRIBUTIONS

José Antonio Sánchez‐Guerrero conceived, designed and performed the study, analysed, wrote, revised and approved the final manuscript. Maria Àngels Cebrià i Iranzo conceived, analysed, wrote, revised and approved the final manuscript. Francisco José Ferrer‐Sargues contributed to the analysis and revised and approved the final manuscript. Sophie Périé conceived, designed, supervised and revised and approved the final manuscript.

## CONFLICTS OF INTEREST

The authors declare that there are no conflicts of interest.

[Correction added on June 1, 2022, after first online publication: Peer review history is not available for this article, so the peer review history statement has been removed.]

## ETHICS STATEMENT

This study was approved by the Commission d'Evaluation et de Recherche Observationnelle en OtoRhinoLaryngologie (CEROL) (Ethics Committee of the Society of Otolaryngology, France). In accordance with French legislation (Public Health Code amended by law no. 2004‐806, 9 August 2004 and the Huriet‐Sérusclat Law 88–1138, 20 December 1988), a patient information letter was issued. Data were strictly anonymous.

## Data Availability

The data that support the findings of this study are available from the corresponding author upon reasonable request.
